# Supplementary material for: Toward conformational identification of molecules in 2D and 3D self-assemblies on surfaces
Source: Commun Chem. 2023 Nov 11;6:246. doi: 10.1038/s42004-023-01036-8 (PMC10640604; doi:10.1038/s42004-023-01036-8)
Supplement: Supplementary file 2 — Supplementary Information [file 42004_2023_1036_MOESM2_ESM.pdf]

Toward conformational identification of molecules in 2D and 3D self-assemblies on surfaces

Ali Hamadeh[1], Frank Palmino[1], Jérémie Mathurin[2], Ariane Deniset-Besseau[2], Louis Grosnit[1], Vincent Luzet[1], Judicaël Jeannoutot[1], Alexandre Dazzi[2], Frédéric Chérioux[1,*]

[1] Université de Franche-Comté, FEMTO-ST, CNRS, F-25000 Besançon, France
[2] Université de Paris-Saclay, Institut de Chimie-Physique, F-91400 Orsay, France

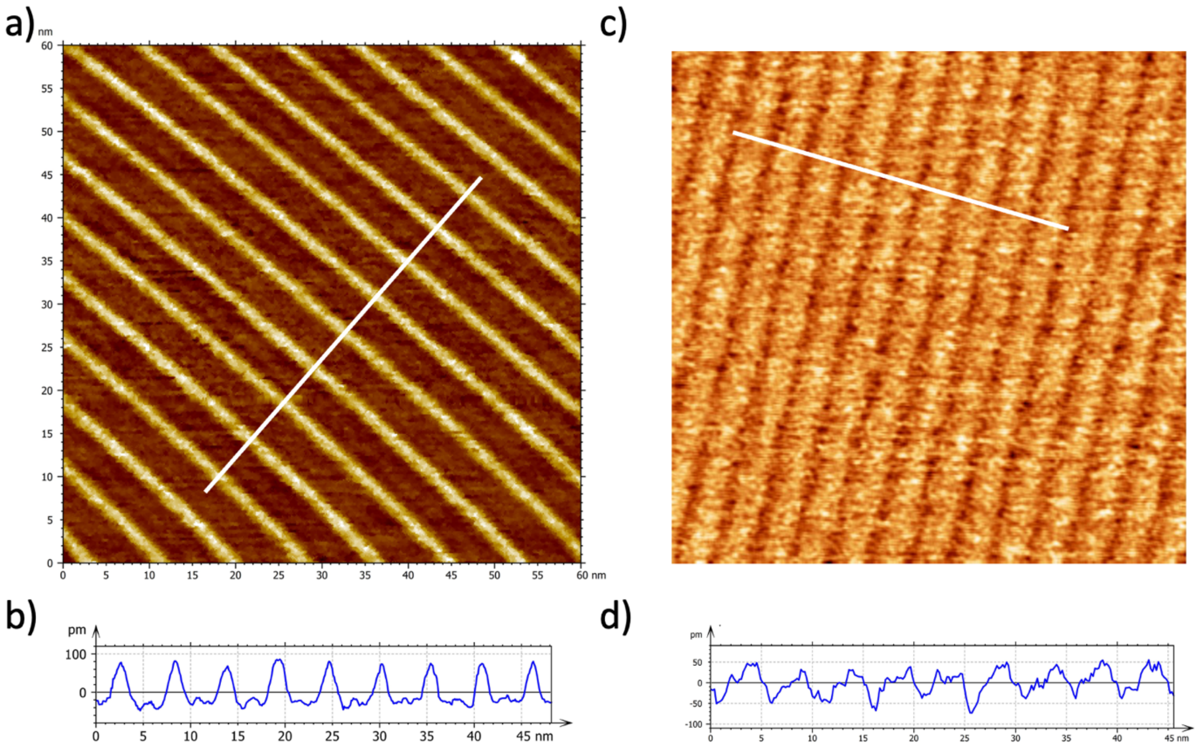

Figure S1. a) STM image (60×60 nm^2, V_s = 1.3 V, I_t = 20 pA, T = RT) of a monolayer of EsterOC18 molecules on a HOPG surface. b) Z-profile taken along the white line of a) highlighting the periodicity of 5.60 nm between two lines. c) Topography AFM (65×65 nm^2) of a monolayer of EsterOC18 molecules on a HOPG surface. d) Z-profile taken along the white line of c) highlighting the periodicity of 5.60 nm between two lines.

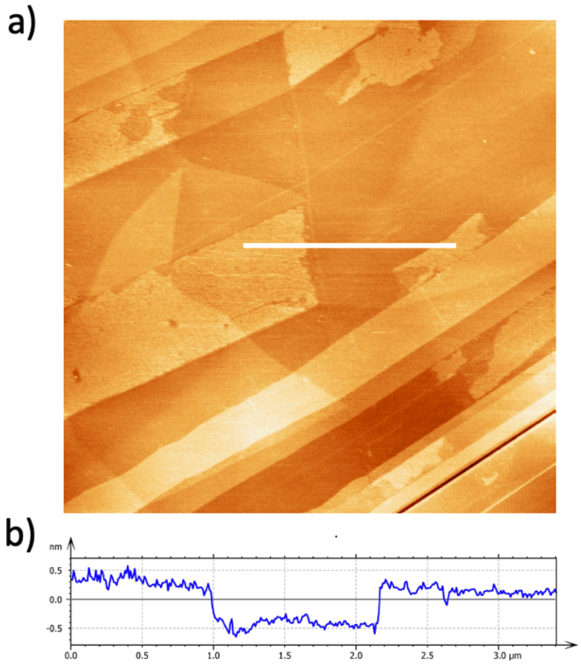

Figure S2. a) Topography AFM (5×5 µm^2) of a monolayer of EsterOC18 molecules on a HOPG surface. b) Z-profile taken along the white line of a) highlighting the monolayer's thickness of 0.8 ± 0.05 nm.

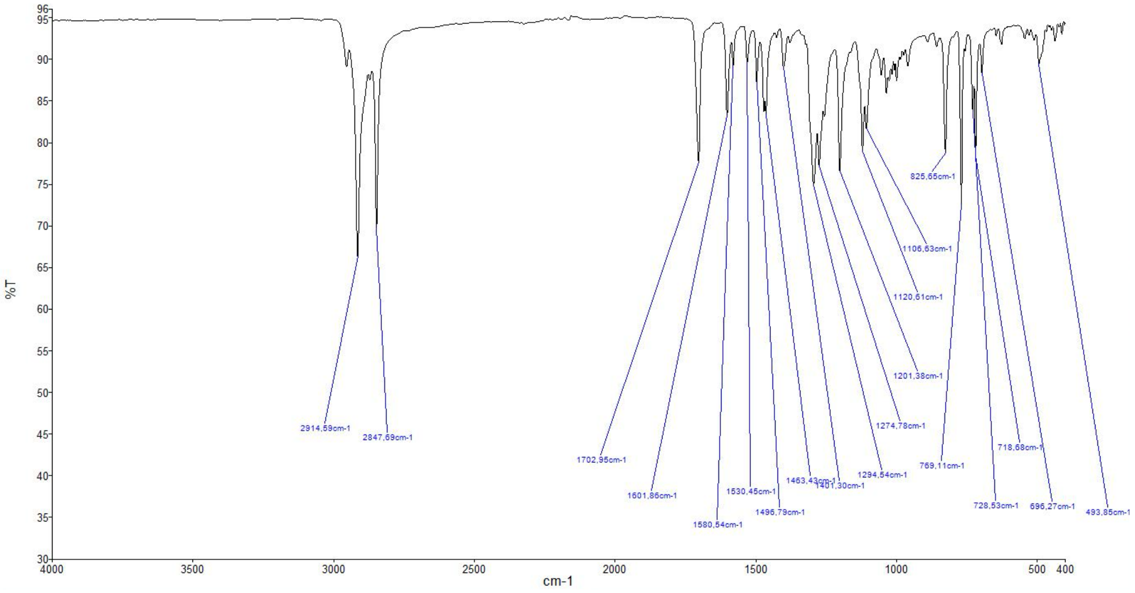

Figure S3. FT-IR spectrum of EsterOC18.

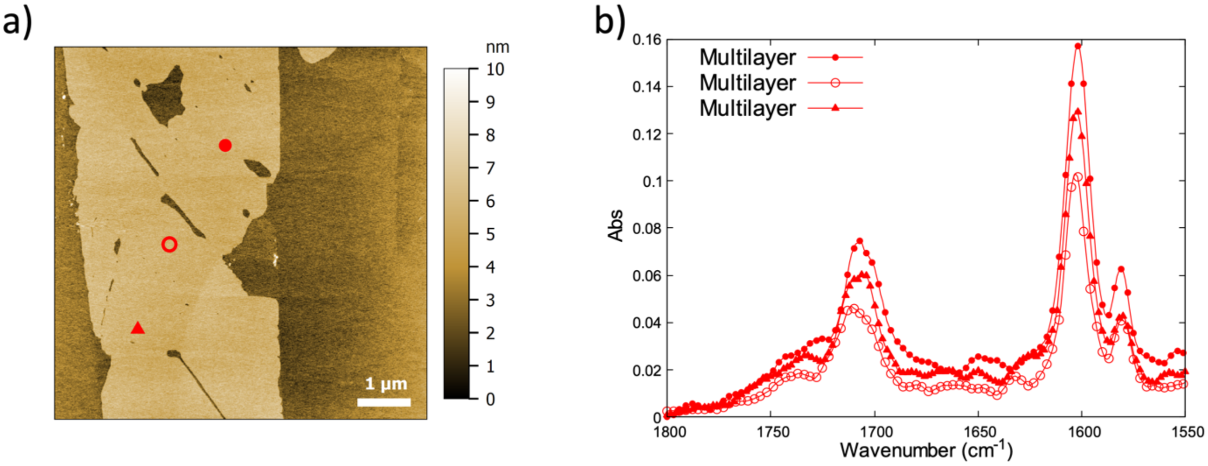

Figure S4. a Large-scale (7×7 µm^2) topography AFM of multilayers (thickness of 3 nm) and monolayer of EsterOC18 on a HOPG surface. The three points selected for the recording of AFM-IR spectra are highlighted in three red forms. b) AFM-IR absorption spectra recorded on the three different points (highlighted in a) of the area covered by multilayers of EsterOC18 molecules.

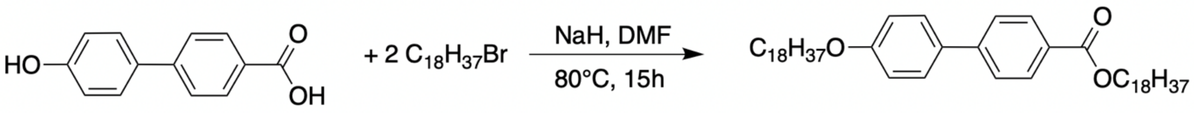

Figure S5. Synthetic pathway of EsterOC18 molecules

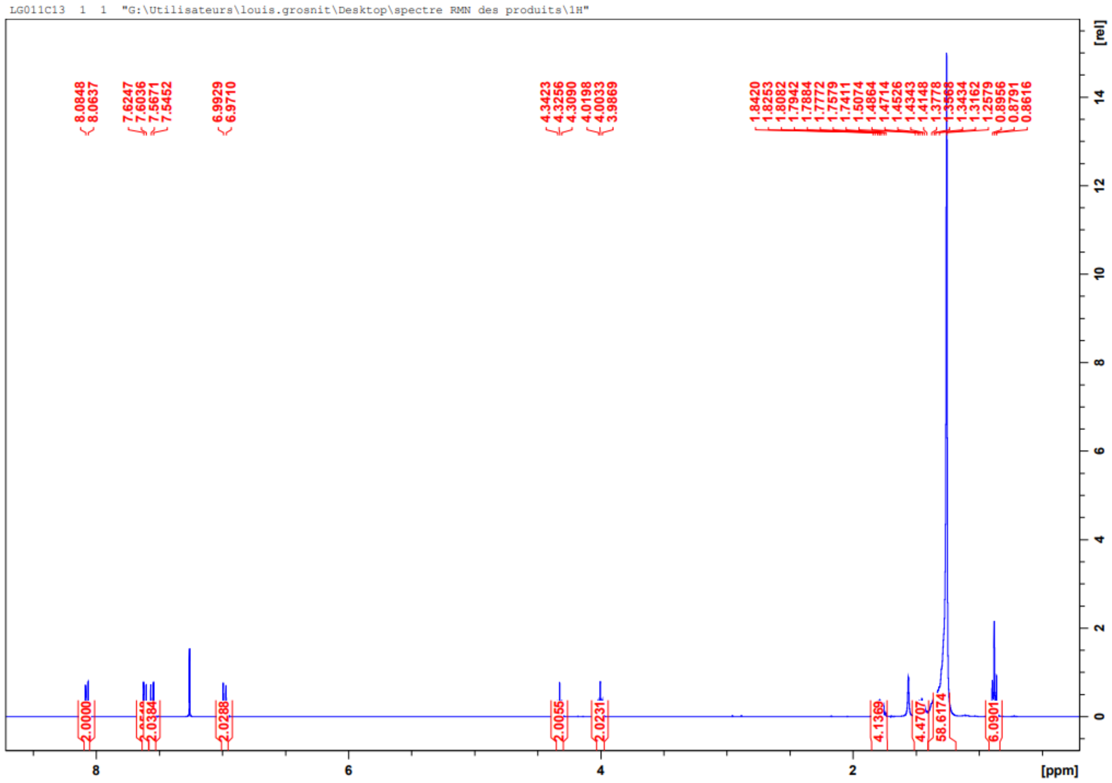

Figure S6. ^{1}H NMR spectrum of EsterOC18 in $CDCl_3$.

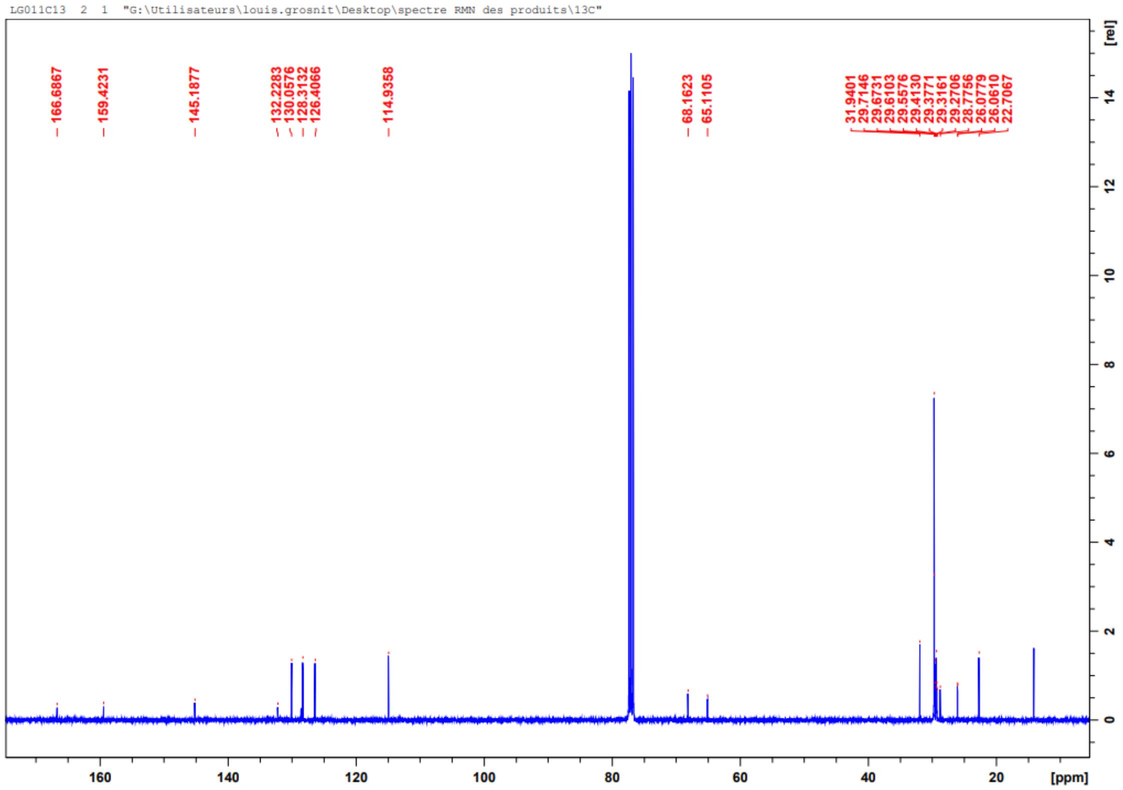

Figure S7. ^{13}C NMR spectrum of EsterOC18 in $CDCl_3$.
